# The European Society of Human Genetics—Young committee- activities and achievements between 2019–2022

**DOI:** 10.1038/s41431-023-01300-4

**Published:** 2023-02-09

**Authors:** Elena Avram, Can Ding, Juliana Xavier de Miranda Cerqueira, Mridul Johari, Ana Raquel Gouveia Freitas da Silva, Ileana-Delia Săbău, Nuru Noor, Silvia Kalantari, Rhys Dore, Rita Barbosa-Matos, Magdalena Mroczek, Francesca Tonini, European Society of Human Genetics - Young Committee, Elena Avram, Juliana Xavier de Miranda Cerqueira, Mridul Johari, Ana Raquel Gouveia Freitas da Silva, Ileana-Delia Săbău, Silvia Kalantari, Rhys Dore, Rita Barbosa-Matos, Magdalena Mroczek, Francesca Tonini

**Affiliations:** 1 Medlife Clinics, Bucharest, Romania; 2 European Society of Human Genetics—Young Committee, Vienna, Austria; 3 Institute of Human Genetics, Universitätsmedizin Mainz, Mainz, Germany; 4 Coeliac Disease Research Center, Faculty of Medicine and Health Technology, University of Tampere, Tampere, Finland; 5 Faculty of Nutrition and Food Sciences, University of Porto, Porto, Portugal; 6 Folkhälsan Research Center, Helsinki, Finland; 7 Department of Medical and Clinical Genetics, Medicum, University of Helsinki, Helsinki, Finland; 8 Serviço de Genética Médica, Departamento de Pediatria, Hospital de Santa Maria, Centro Hospitalar Universitário Lisboa Norte, Lisboa, Portugal; 9 Personal Genetics, Bucharest, Romania; 10 Cambridge University Hospitals, Cambridge, UK; 11 Department of Medical Sciences, University of Turin, Turin, Italy; 12 Barts Health NHS Trust, London, UK; 13 i3S—Instituto de Investigação e Inovação em Saúde, Universidade do Porto, Porto, Portugal; 14 Center for Cardiovascular Genetics & Gene Diagnostics, Foundation for People with Rare Diseases, 8952 Schlieren-Zurich, Switzerland; 15 Cambridge Genomics Laboratory, Addenbrooke’s Hospital, Cambridge University Hospitals NHS Foundation Trust, Cambridge, UK

**Keywords:** Health policy, Genetics research

## Abstract

The European Society of Human Genetics—Young Committee (ESHG-Y) aims to support young human geneticists by developing strategies and programs for better education and creating a strong network in all European countries. In this report, we present the ESHG-Y projects conducted since its conception. We organized the educational sessions at the ESHG Annual Conference, the European Dysmorphology Meetings, and a virtual session in collaboration with the European Board of Medical Genetics (EBMG). Also, the ESHG-Y regularly promotes relevant activities and succeeded in creating an active network of young geneticists. Our representatives have a supportive role in well-known organizations such as: ESHG Board, ESHG Scientific Program Committee, ESHG Education Committee, EBMG, ERN-Ithaca, Unique - Rare Chromosome Disorder Support group, Orphanet, EuroGEMS, MOOC BIG and more. Taking into consideration all activities and ongoing projects, we can state that the ESHG-Y successfully achieves its objectives and brings young professionals together.

## Introduction

The European Society of Human Genetics—Young (ESHG-Y) was launched with great enthusiasm as a committee, in June 2019 by human genetic trainees with common goals and a shared vision regarding human genetics [1, 2]. This occurred under the guidance of the European Society of Human Genetics (ESHG).

**Fig. 1:**
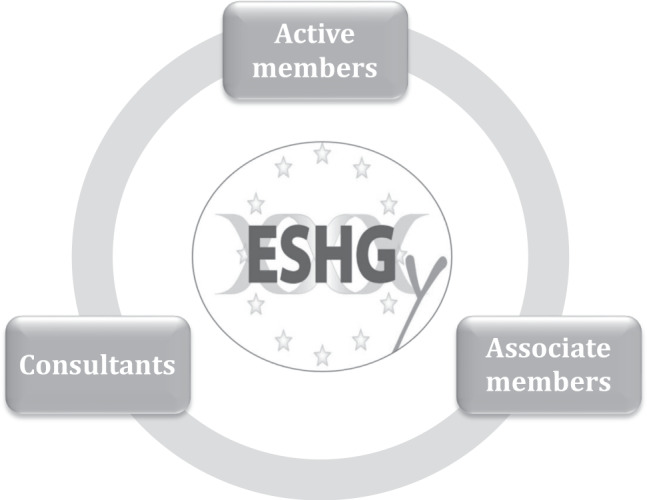
ESHG-Y Structure. The ESHG-Y has 3 type of members: active members, consultants, associate members.

In the last year, significant adjustments have been made and currently, the structure of the committee is composed of different types of members (Fig. 1):

- “Active members” are elected by all trainee members of the ESHG for a period of 2 years (one chair, two vice-chairs, two secretaries, and two spokespersons) and are responsible for all decisions taken by the committee;
- “Consultants” are elected based on the request of the committee from former ESHG-Y Committee members; additionally they are not allowed to vote and their role is to supervise ongoing projects;
- “Associate members” are invited directly by the ESHG-Y due to the individual’s outstanding Curriculum Vitae and achievements to fill different skill gaps and are not allowed to vote.

In addition, the committee grants the position of “Officer” to any volunteer that is involved in our projects.

**Fig. 2:**
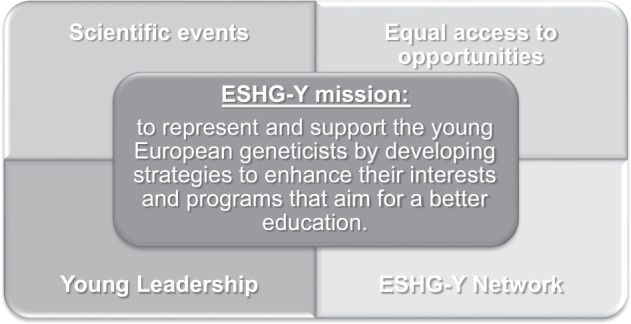
ESHG-Y Mission and objectives. ESHG-T mission: represent and support young European human geneticists. ESHG-Y objectives: organize scientific events, ensure equal access to educational opportunities, acreate a professional network and support junior human geneticists to become leaders.

ESHG-Y’s initiative allows trainees to have a significant impact by creating a strong network between all European countries and sharing professional experiences. Our mission is to represent and support the young European geneticists by developing strategies and programs that aim for a better education. We intend to create and implement new projects that will enhance the performance of young geneticists and sustain them to be heard, understood, and empowered. The efforts of our committee are centered on four main objectives: organize scientific events, ensure equal access to educational opportunities, create a professional network, and supporting junior geneticists to become leaders (Fig. 2).

Since our commencement in 2019, we have engaged with different entities and developed multiple projects that are in accordance with our mission and objectives. In the last 4 years, partnerships with highly regarded organizations have continued to grow, maintaining their positive long-term trend.

## Organizing scientific events

Our main focus is to invest energy and resources into creating compelling educational opportunities for the young generation of human geneticists. Relevant scientific events to which we contributed and had a decisive role were: the ESHG Annual Conference and the European Dysmorphology Meeting.

The ESHG-Y organized a workshop focusing on professional development during the 2020 ESHG Annual Conference. Ever since then, we have organized an annual scientific session addressed to the young generation of geneticists. We participated with three posters at this conference between 2020–2022 (Table 1). Additionally in 2022 in Vienna, we had the opportunity to have our own lounge at the ESHG Conference, where we could promote our committee and present projects.

**Table 1:** ESHG-Y at the ESHG Conference 2020–2022.

	Topic	Type	Speakers / Panel experts/ Authors
2020	ESHG Young Board and Young Geneticist Network - Professional Development for the Next Generation • Work-life balance: how to have it all • ERN: Opportunities for the next generation • Public choice - Human Genetics in different countries	Workshop	Marni J. Falk, Alain Verloes, Sofia Douzgou
	The Young Geneticists Network: using social media to unite younger trainees in Human Genetics around the world	Poster	Florence Riccardi, Celia Soares, Ruta Marcinkute, Patricia Calapod
2021	ESHG-Y: Human organoids as genetic disease models • Human stem cells-based organoids for personalized disease modeling in human genetics • Modeling human lung development and disease using hPSC-derived organoids	Educational session	Hans Clevers, Hans - Willem Snoeck
2022	ESHG-Y: Filling the gaps by publishing negative results	Educational session	Virginia Arechavala-Gomez, Devang MehtaAlisdair McNeill, Magdalena Skipper
	The emerging role of the European Society of Human Genetics-Young Committee	Hybrid poster	Elena Avram, Juliana Xavier de Miranda Cerqueira, Can Ding, Mridul Johari, Ana Raquel Gouveia Freitas da Silva, Ileana - Delia Săbău, Nuru Noor
	Worldwide use of EuroGEMS.org, the ESHG’s guide to online educational resources, and its new full Spanish translation	Hybrid Poster	Adam P. Tobias, Irene Esteban, Anna Esteve Garcia, Elena Avram, Ana Raquel Gouveia Freitas Da Silva, Juliana Xavier de Miranda Cerqueira, Edward S. Tobias

Starting in 2021, two ESHG-Y members have been involved in organizing the annual European Dysmorphology Conference which is held by ERN-ITHACA.

## Advocating for equal access to educational opportunities

All junior geneticists have the right to equal access to education. However, due to limited access to infrastructure, knowledge, or human resources, becoming a well-trained geneticist can be challenging in a developing country. ESHG-Y tries to fill this gap by developing collaborations with important entities that offer educational opportunities in the field of human genetics.

From 2020 ERN-ITHACA has offered the ESHG-Y the opportunity to contribute with presentations at their biannual ITHACA Board Meeting; subsequently, collaborations with the EuroDysmorpho Meeting and UNIQUE were successfully established.

UNIQUE is a registered charity that offers support and knowledge to families and caregivers of patients affected by a rare chromosome disorder; more than that it provides a network for patients and professionals. UNIQUE and ERN-ITHACA aim to offer accurate and simplified information on rare genetic disorders for non-native English-speaking families. For this purpose, the ESHG-Y has coordinated since March 2021 the recruitment of native speakers of different languages on a voluntary basis as proofreaders for the UNIQUE automated translated guides. The volunteers were recruited from the European Young Geneticist Network (YGN). We have since succeeded in enrolling 32 volunteers that contributed with translations in French, German, Italian, Lithuanian, Polish, Portuguese and Spanish.

ESHG-Y also collaborated with the European Joint Program on Rare Disease (EJP RD) by providing support with the development of the “Diagnosing Rare Diseases” MOOC for which we recruited six beta-testers and five mentors in 2021.

Also in 2021, our committee was offered the opportunity to have a liaison member in the European Board of Medical Genetics. On the 1^st^ of December 2021, together with Dr. Jonathan Berg (Chair of the ECMGG Exam Committee) and Dr. Laura Pölsler (Secretary of the ECMGG Exam Committee), we organized a special virtual session called “Becoming European Board Certified in Medical Genetics and Genomics”. The discussion aimed to highlight the importance of holding the European Certification in Medical Genetics and Genomics (ECMGG) degree and to provide essential information about this exam.

DNA Day, April 25th, is commemorated internationally as a celebration of genetics. The DNA Day Essay and Video contest, supervised by Dr. Christophe Cordier, intends to challenge European high school students to reflect on the importance of human genetics. The ESHG-Y committee became involved as judges for the first time in this event in 2021, providing support with the evaluation of the essays and videos. The topic suggested by us, “How can DNA help us to discover ancient human history?” was elected for 2022.

The EuroGEMS website provides impressive digital educational resources related to genetics and genomics. The online material is addressed to genetic specialists, non-genetics professionals, students and the public [3, 4]. The ESHG-Y Committee had a supportive role by contributing to the ongoing EUROGEMs.org translation into Portuguese and French which will increase access from Portuguese and French-speaking countries.

Since 2021, we have had two representatives in the ESHG Education Committee (ESHG EduComm) to support their projects. The ESHG International Mentorship Programme annually provides five young professionals the opportunity to undertake a 1 week educational and observational placement within a European genetic institution. The Programme was launched in 2022 and received applications from different continents. Members from the ESHG-Y were involved in the election process of the awardees and had a supportive role.

In 2022, the ESHG’s Podcast “Genetic Sounds”, coordinated by Dr. Sofia Douzgou, was launched with great excitement. This podcast discusses different topics in genetics that involve us all and is addressed to both professionals and non-professionals. Current and former ESHG-Y representatives were invited to make significant contributions in the 2nd episode “Let’s talk about sex in genetics” and the 3rd episode “Let’s talk about access to genetic services”.

In addition to these partnerships, in the future the committee will also seek to produce and disseminate biannual newsletters, to keep young members of the ESHG updated on upcoming training courses, events, and development opportunities.

## Creating a professional network of young human geneticists

Having the opportunity to share experiences and opportunities between developed and developing countries in Europe will surely lead to important progress in the field of human genetics and will inspire and engage young geneticists to reshape the future of human genetics.

The ESHG-Y committee is present on social media through an active Facebook page and a Twitter channel (@eshg_young), both used for disseminating information regarding our projects. Furthermore, since 2019, the ESHG-Y manages the Young Geneticist Network (YGN) which aims to allow its members to share and discuss information related to human genetics [2]. The ESHG-Y Twitter account, created in August 2021, has additionally served as a network engagement boost of the European and International young geneticists on social media.

To further promote our activities a joint manuscript (2022) from the YGN and ESHG-Y Committee was published in the European Journal of Human Genetics, a journal with broad international readership [2]. This helped to highlight, together with a previous publication from ESHG (2021) that included members of the ESHG-Y Committee, the importance of our organization in delivering genomic education and promoting the young generation [4].

In 2022, the ESHG-Y was invited to summarize its accomplishments in the ESHG Newsletter, this way highlighting the importance of involving the young generation in such organizations.

For 2023 our Committee has been invited to offer support for the Young Investigator Forum (ICHG2023 Meeting) affiliated to the African Society of Human Genetics (AfSHG).

## Supporting young geneticists to become leaders

Having good leadership that promotes the ESHG-Y’s mission, provides direction and is crucial for engaging the young generation in projects that aim to equally develop the genetic field in Europe and beyond.

In order to represent the young human geneticists’ interests representatives from the ESHG-Y are involved in different committees like the ESHG Board, ESHG Scientific Program Committee (SPC), ESHG Education Committee (EduCom), ESHG Social Media Committee and European Board of Medical Genetics (EBMG). All these committees have the purpose to organize and promote different scientific events and facilitate access to a diverse portfolio of genetic courses and educational material.

In 2022, the ESHG Journal offered section editor and editorial board positions for ESHG-Y representatives, while the ESHG Public and Professional Policy Committee (PPPC) offered us a permanent position as an observer.

Former, current, and future members of the ESHG-Y gain experience and notability by being involved in the ESHG-Y Committee, whilst also developing friendships and collaborations with renowned geneticists from Europe. This allows them to become leaders and forces of change in their own national scientific communities and to augment the development of the human genetics field within their own countries.

## Perspectives

For the near future, we will continue to promote ESHG-Y’s activities and attract new members and we would like to create a network of young geneticist committees in all European countries. We will be seeking to develop further activities:

- The “ESHG Observership for Young Geneticists” program (first edition in 2023) offers annual financial support (2000 euro) for five young human geneticists who wish to observe specific areas of clinical care or laboratory techniques at highly regarded European human genetics departments for a maximum period of 1 month;
- The “ESHG-Young European Committees Group” encourages young geneticist to organize in formal committees associated to European human genetic professional organizations, and aims to connect them and develop a strong network;
- The “ESHG-Young International Leadership Group” has the objective to build a strong partnership with all young international professional organizations and work together on projects; furthermore one of its main activities is to co-create virtual sessions regarding common topics periodically.

Moreover, we will continue our established collaborations and partnerships and we aim to engage many more young geneticists focusing on the opportunities and advancements in European genetic education.

## Conclusion

In the last 4 years, the ESHG-Y has succeeded in achieving its objectives by developing ongoing projects and partnerships. With the growth and expansion of the genetic field comes the need to inspire, interconnect and encourage the younger generation to have a major professional impact on the equitable development of this speciality in Europe.
